# Prognostic Value of D-dimer to Lymphocyte Ratio (DLR) in Hospitalized Coronavirus Disease 2019 (COVID-19) Patients: A Validation Study in a National Cohort

**DOI:** 10.3390/v16030335

**Published:** 2024-02-22

**Authors:** Crhistian-Mario Oblitas, Pablo Demelo-Rodríguez, Luis-Antonio Alvarez-Sala-Walther, Manuel Rubio-Rivas, Francisco Navarro-Romero, Vicente Giner Galvañ, Lucía de Jorge-Huerta, Eva Fonseca Aizpuru, Gema María García García, José Luis Beato Pérez, Paula María Pesqueira Fontan, Arturo Artero Mora, Juan Antonio Vargas Núñez, Nuria Ramírez Perea, José Miguel García Bruñén, Emilia Roy Vallejo, Isabel Perales-Fraile, Ricardo Gil Sánchez, José López Castro, Ángel Luis Martínez González, Luis Felipe Díez García, Marina Aroza Espinar, José-Manuel Casas-Rojo, Jesús Millán Núñez-Cortés

**Affiliations:** 1 Internal Medicine Department, Hospital Clínico de Santiago, 15706 Santiago de Compostela, Spain; 2 Sanitary Research Institute of Santiago, 15706 Santiago de Compostela, Spain; 3 School of Medicine, Universidad Complutense de Madrid, 28040 Madrid, Spain; pbdemelo@hotmail.com (P.D.-R.);; 4 Internal Medicine Department, Hospital General Universitario Gregorio Marañón, 28007 Madrid, Spain; 5 Sanitary Research Institute Gregorio Marañón, 28009 Madrid, Spain; 6 Internal Medicine Department, Hospital Universitario de Bellvitge, 08908 Barcelona, Spain; 7 Internal Medicine Department, Hospital Costa del Sol, 29603 Marbella, Spain; f.navarroromero@gmail.com; 8 Internal Medicine Department, Hospital Universitario San Juan de Alicante, 03550 Alicante, Spain; 9 Internal Medicine Department, Hospital Universitario 12 de Octubre, 28041 Madrid, Spain; luciajorgehuerta@hotmail.com; 10 Internal Medicine Department, Hospital de Cabueñes, 33394 Gijon, Spain; evamfonseca@yahoo.es; 11 Internal Medicine Department, Complejo Hospitalario Universitario de Badajoz, 06010 Badajoz, Spain; geminway21@hotmail.com; 12 Internal Medicine Department, Complejo Hospitalario Universitario de Albacete, 02006 Albacete, Spain; 13 Internal Medicine Department, Hospital Universitario Dr. Peset, 46017 Valencia, Spain; arturo.artero@uv.es; 14 Internal Medicine Department, Hospital Universitario Puerta de Hierro Majadahonda, 28222 Madrid, Spain; 15 Internal Medicine Department, Hospital General Universitario de Elda, 03600 Alicante, Spain; 16 Internal Medicine Department, Hospital Miguel Servet, 50009 Zaragoza, Spain; 17 Internal Medicine Department, Hospital Universitario La Princesa, 28006 Madrid, Spain; 18 Internal Medicine Department, Hospital Infanta Sofía, 28702 Madrid, Spain; 19 Internal Medicine Department, Hospital Universitario La Fe, 46026 Valencia, Spain; 20 Internal Medicine Department, Hospital Público de Monforte de Lemos, 27400 Lugo, Spain; jose.lopez.castro@sergas.es; 21 Internal Medicine Department, Complejo Asistencial Universitario de León, 24008 Leon, Spain; 22 Internal Medicine Department, Hospital Torrecardenas, 04009 Almeria, Spain; 23 Internal Medicine Department, Hospital Insular de Gran Canaria, 35016 Las Palmas de Gran Canarias, Spain; 24 Internal Medicine Department, Infanta Cristina University Hospital of Parla, 28981 Madrid, Spain; 25 Sanitary Research Institute Puerta de Hierro—Segovia de Arana, 28222 Madrid, Spain

**Keywords:** biomarkers, D-dimer to lymphocyte ratio, COVID-19, mortality, ratios, SARS-CoV-2

## Abstract

Background: This study aimed to validate the role of the D-dimer to lymphocyte ratio (DLR) for mortality prediction in a large national cohort of hospitalized coronavirus disease 2019 (COVID-19) patients. Methods: A retrospective, multicenter, observational study that included hospitalized patients due to SARS-CoV-2 infection in Spain was conducted from March 2020 to March 2022. All biomarkers and laboratory indices analyzed were measured once at admission. Results: A total of 10,575 COVID-19 patients were included in this study. The mean age of participants was 66.9 (±16) years, and 58.6% (6202 patients) of them were male. The overall mortality rate was 16.3% (*n* = 1726 patients). Intensive care unit admission was needed in 10.5% (*n* = 1106 patients), non-invasive mechanical ventilation was required in 8.8% (*n* = 923 patients), and orotracheal intubation was required in 7.5% (789 patients). DLR presented a c-statistic of 0.69 (95% CI, 0.68–0.71) for in-hospital mortality with an optimal cut-off above 1. Multivariate analysis showed an independent association for in-hospital mortality for DLR > 1 (adjusted OR 2.09, 95% CI 1.09–4.04; *p* = 0.03); in the same way, survival analysis showed a higher mortality risk for DLR > 1 (HR 2.24; 95% CI 2.03–2.47; *p* < 0.01). Further, no other laboratory indices showed an independent association for mortality in multivariate analysis. Conclusions: This study confirmed the usefulness of DLR as a prognostic biomarker for mortality associated with SARS-CoV-2 infection, being an accessible, cost-effective, and easy-to-use biomarker in daily clinical practice.

## 1. Introduction

Infection by the severe acute respiratory syndrome coronavirus 2 (SARS-CoV-2) causes the coronavirus disease 2019 (COVID-19), which has become the worst pandemic worldwide in the last few decades, with officially reported deaths exceeding 6.8 million, which are likely underestimated [1,2,3,4,5]. Although the SARS-CoV-2 virus presents a substantial pulmonary burden producing hypoxemic respiratory failure and acute respiratory distress syndrome (ARDS), extrapulmonary complications have also been reported [6]. Pathophysiology seems to be complex; however, some clues have been elucidated. This virus appears to enter the epithelial cells of the respiratory tract by binding to the angiotensin-converting enzyme 2 (ACE2) receptor, which is widespread in various organs including the respiratory system. Thus, the virus and the down-regulation of ACE2 leads to direct cytotoxic damage and a pro-inflammatory state, commonly misnamed as a “cytokine storm” [4,7]. Broadly, 14–20% of patients required admission to the intensive care unit (ICU), with an overall mortality of around 2%. In addition, among those admitted to an ICU, the mortality dramatically increases up to nearly 50% [1,2,3,8]. In this setting, it has been described that the presence of previous comorbidities and laboratory biomarkers, such as low lymphocyte count, high levels of D-dimer, C-reactive protein (CRP), and other inflammatory biomarkers, alongside some laboratory indices (or ratios), is associated with poor prognosis in SARS-CoV-2 infection [1,4,9,10,11,12]. Previous works have proposed that combining two different biomarkers in ratios might improve their prognostic capacity when compared to the biomarkers themselves. Previously, we hypothesized that SARS-CoV-2 might induce endothelial damage (vascular dysfunction) and activate the immune system, and those factors provoked a disbalance, provoking a pro-inflammatory (low lymphocyte count) and pro-coagulant state (elevation of D-dimer serum levels) [8,11,12,13,14,15,16]; taking into account the same theoretical basis, our working group evaluated, in a single-center cohort of 1113 hospitalized COVID-19 patients, the role of 10 laboratory indices as prognostic biomarkers, including both well-known ratios described previously in the literature (neutrophil to lymphocyte ratio (NLR), derived neutrophil to white blood cells minus neutrophile ratio (d-NLR), platelet to lymphocyte ratio (PLR), CRP to lymphocyte ratio, and CRP to albumin ratio) and proposed new laboratory indices. After multivariate regression logistic analysis, only the new proposed ratio, the D-dimer to lymphocyte ratio (DLR), showed an independent association in terms of mortality (adjusted OR 2.33; *p* < 0.05) [12].

This study aims to validate the usefulness of the novel D-dimer to lymphocyte ratio for in-hospital mortality prediction in a large national cohort of hospitalized COVID-19 patients.

## 2. Materials and Methods

### 2.1. Study Design

The present study is a retrospective, multicenter, observational study that includes patients hospitalized due to SARS-CoV-2 infection from March 2020 to March 2022 in Spain. The Spanish Society of Internal Medicine is the sponsor of the nationwide SEMI-COVID-19 Registry.

### 2.2. Population of the Study

**Figure 1:**
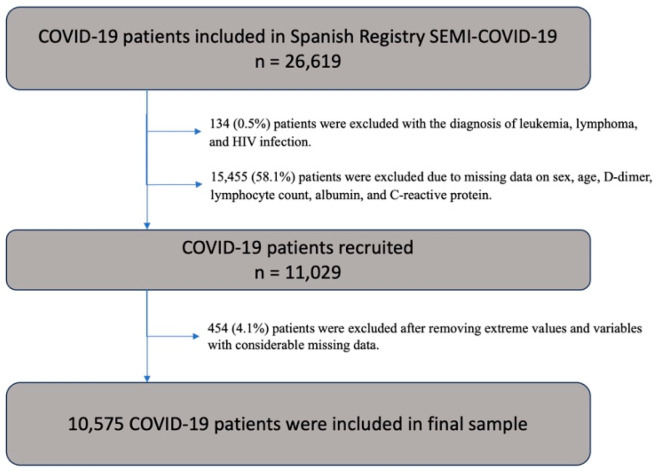
Patient inclusion flowchart. HIV denotes human immunodeficiency virus.

For the SEMI-COVID-19 Registry, an online electronic data capture system (DCS) was developed, which includes a database manager along with procedures for the verification of data and contrasting information against the original medical record to ensure the best possible quality of data collection. Patient-identifiable data were dissociated and anonymized. Data were collected retrospectively and included more than 300 variables grouped under different headings: inclusion criteria, epidemiological data, personal medical and medication history, symptoms and physical examination findings at admission, wide laboratory panel at admission, diagnostic imaging tests, and complications during the hospitalization. More in-depth information about the registry can be found in previously published works [14]. The inclusion criteria were age over 18 years old, consent information, and complete data for the outcome variable. The exclusion criteria for baseline characteristics were the presence of leukemia, lymphoma, and infection by the human immunodeficiency virus, since most cellular indices are based on lymphocyte count. Patients without registered D-dimer, lymphocyte, CRP, albumin, and/or white cell count values at admission were excluded. From 28,444 patients included in the SEMI-COVID-19 Registry in March 2022, a total of 11,029 patients were recruited from the database. The final sample included 10,575 patients after removing extreme values for each quantitative variable and variables with considerable missing data and those of in-hospital patients before statistical analysis (Figure 1). We analyzed demographic characteristics, clinical and radiological presentation, and laboratory biomarkers on admission and treatments. The outcome was in-hospital mortality. The laboratory indices that were measured included neutrophil to lymphocyte ratio (NLR), derived neutrophil to white blood cells minus neutrophile ratio (d-NLR), platelet to lymphocyte ratio (PLR) (10^6^/L), CRP to lymphocyte ratio (mg/L)/(10^6^/L), CRP to albumin ratio (mg/L)/(g/dL), and the new D-dimer to lymphocyte ratio (ng/mL)/(10^6^/L). All biomarkers and laboratory indices analyzed were measured once at admission.

### 2.3. Data Analysis

The Shapiro–Wilk test determined the normality of continuous quantitative variables. This study reported categorical data as proportions and continuous data as mean and standard deviation (SD) or median and interquartile range (IQR), depending on their normality. The Student’s *t*-test and analysis of variance (ANOVA) were performed for normally distributed variables, while the Mann–Whitney U and the Kruskal–Wallis test were used for non-normally distributed variables. The Kaplan–Meier (KM) estimator was used to graphically represent the event death, and the Cox regression test was used to evaluate the hazard ratio (HR). The receiver operating characteristic area under the curve (AUC) analysis and the Youden index were used to explore the best cut-off point for predicting mortality for laboratory biomarkers. The univariate logistic regression test evaluated the association between the different variables and outcome (crude odds ratio (OR)). The multivariate logistic (adjusted OR) regression analysis included adjustment for potential confounding variables and included variables with *p* < 0.1 and a strong association in the univariate analysis. All tests were two-sided, and the level of statistical significance was set at *p* < 0.05. The statistical analysis was carried out by using R, version 4.2.2, R Core Team, Vienna, Austria (main R packages used: tidyverse, pROC, survival).

## 3. Results

A total of 10,575 COVID-19 patients were included in this study. The mean age of participants was 66.9 (±16) years, and 58.6% (6202 patients) of them were male. Among the comorbidities observed, arterial hypertension was the most common, affecting 51.8% of the patients (*n* = 5474), followed by dyslipidemia in 40.3% (*n* = 4259 patients), diabetes in 22.9% (*n* = 1098 patients), atrial fibrillation in 10.3% (*n* = 1089 patients), and dementia in 7.8% (*n* = 815 patients). The overall mortality rate was 16.3% (*n* = 1654 patients). In addition, ICU admission was needed in 10.5% (*n* = 1106 patients), non-invasive mechanical ventilation was required in 8.8% (*n* = 923 patients), and orotracheal intubation was required in 7.5% (789 patients). The median length of hospitalization was 9 days (6–15). Table 1 describes and summarizes the baseline characteristics of the total sample, while a comparison of distribution frequency between survivors and non-survivors is shown.

**Figure 2:**
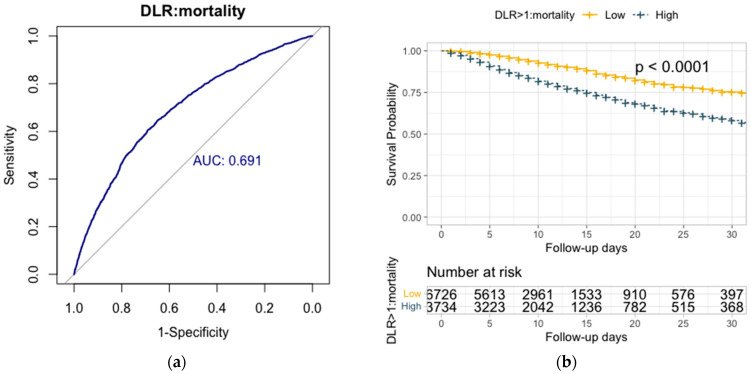
(**a**) showed the predictive capacity for in-hospital mortality of D-dimer to lymphocyte ratio (DLR) showed a ROC area under the curve of 0.69 (95% CI, 0.68–0.71). (**b**) showed overall survival Kaplan–Meier analyses of in-hospital mortality for DLR > 1 stratified according to the optimal cut-off, showing a hazard ratio of 2.24 (95% CI 2.03–2.47). The absolute numbers of surviving patients on days 0, 5, 10, 15, 20, 25, and 30 comparing levels from above or below the optimal cut-off.

The predictive capacity of the D-dimer to lymphocyte ratio demonstrated an AUC of 0.69 (95% CI, 0.68–0.71) for in-hospital mortality (Figure 2, panel A). DLR > 1 was identified as the optimal cut-off point for mortality, with a negative predictive value (NPV) of 90%. NLR exhibited an AUC of 0.67 (95% CI, 0.65–0.68) for in-hospital mortality, with an optimal cut-off above 6.5 for predicting mortality (NPV 88%). The predictive capacity of d-NLR revealed an AUC of 0.66 (95% CI, 0.65–0.68) for in-hospital mortality. d-NLR > 4 was the optimal cut-off for mortality (NPV 89%). PLR showed an AUC of 0.58 (95% CI, 0.57–0.6) for in-hospital mortality, with an optimal cut-off above 290. The predictive capacity of the CPR to lymphocyte ratio showed an AUC of 0.67 (95% CI, 0.66–0.69) for in-hospital mortality, with an optimal cut-off above 0.1 for mortality (NPV 89%). The CPR to serum albumin ratio showed an AUC of 0.67 (95% CI, 0.65–0.68) for in-hospital mortality (Figure 2a), with an optimal cut-off above 25 for mortality (NPV 90%).

Univariate logistic regression analysis for in-hospital mortality showed the following: DLR > 1 (crude OR 3.54; 95% CI 2.34–5.47), NLR > 6.5 (crude OR 3.58; 95% CI 2.39–5.44), d-NLR > 4 (crude OR 3.58, 2.40–5.39), CRP to lymphocyte ratio > 0.1 (crude OR 3.41, 95% CI 2.26–5.25), and CRP to serum albumin ratio > 25 (crude OR 2.93; 95% CI 1.96–4.43). On the other hand, the multivariate analysis presented an independent association with in-hospital mortality for DLR > 1 (adjusted OR 2.12, 95% CI 1.119–4.08; *p* = 0.02), age over 70 (adjusted OR 2.63; 95% CI 1.60–4.41; *p* =< 0.01), and ARDS (adjusted OR 10.96; 95% CI 5.69–23.46; *p* < 0.01). Table 2 summarizes the association for univariate and multivariate analysis related to mortality.

Survival analysis for in-hospital mortality showed DLR > 1 (HR 2.24; 95% CI 2.03–2.47; *p* < 0.01), NLR > 6.5 (HR 1.92; 95% CI 1.74–2.11; *p* < 0.01), d-NLR > 4 (HR 1.98; 95% CI 1.80–2.18; *p* < 0.01), CRP to lymphocyte ratio > 0.1 (HR 1.97; 95% CI 1.78–2.17; *p* < 0.01), and CRP to serum albumin ratio > 25 (HR 1.89; 95% CI 1.71–2.09; *p* < 0.01). The survival Kaplan–Meir curve for in-hospital mortality and DLR cut-off above 1 is shown in Figure 2b.

## 4. Discussion

Since the presence of a low lymphocyte count and elevated D-dimer levels seems to be associated with poor prognosis in SARS-CoV-2 infection, searching for laboratory biomarkers presents a great clinical interest [15,16,17]. In this setting, Zhang H et al. [18] conducted a meta-analysis with 3337 patients and found that patients with severe COVID-19 had lower levels of lymphocytes, with a weight mean difference (WMD) of −0.468 (×10^9^/L) (95% CI; −0.543 to −0.394), but higher levels of NLR, with a WMD of 6.939 (95% CI 4.581–9.297) and D-dimer levels with a WMD of 1.217 mg/L (95% CI 0.788–1.646), when compared with non-severe COVID-19. Another meta-analysis on 61 studies (15,522 patients) found that higher levels of NLR were associated with all-cause mortality in hospitalized COVID-19 patients in the univariate analysis but not in the multivariate analysis (crude OR 12.6; 95% CI 6.88–23.06), showing a high degree of heterogeneity (I2 = 98%) [19]. Contrarily, our working group found no association between NLR and 30-day mortality (crude OR 1.03; 95% CI 0.99–1.05) in a prospective cohort of 95 critically ill patients admitted to the ICU [17]. Further, some other laboratory indices were also evaluated. Sarkar et al. [20] evaluated the role of PLR in COVID-19 patients upon admission (14 studies; 2768 patients) for mortality, showing a WMD of 66.10 (95% CI: 47.75–84.44) with high heterogeneity (I2 = 89%) and low-quality evidence; a meta-analysis of 71 studies found no association for PLR with a poor prognosis in terms of mortality [21]. Moreover, a retrospective study evaluated the role of the CRP to serum albumin ratio in 272 COVID-19 patients in terms of mortality, finding an optimal cut-off above 21.5 (c-statistic of 0.72; 95% CI 0.66–0.77) [22], whereas another study that involved 3471 patients showed an optimal cut-off above 25 to be associated with mortality (OR, 1.47; 95% CI 1.19–1.82) [23]. A meta-analysis conducted by Zavalaga-Zegarra et al. [24] evaluated the potential association of the CPR to albumin ratio in terms of mortality in 17 studies (7164 COVID-19 patients), finding higher values in non-survivors (MD: 2.59; 95% CI: 1.95–3.23) with severe heterogeneity (I2 = 92%). A sensitivity analysis that included studies with a low risk of bias (5 studies) showed a significant association (MD: 2.99; 95% CI: 2.47–3.51; I2 = 0%), where cut-off values were only stated in two studies without specifying units of measure.

Formerly, our working group evaluated different laboratory indices published in the literature and a new index called the D-dimer to lymphocyte ratio as a potential prognostic biomarker in a single-center cohort of 1113 hospitalized COVID-19 patients. DLR > 0.6 showed an independent association for 30-day mortality (adjusted OR 2.33; 95% CI 1.01–5.38), but no other laboratory indices showed any association in multivariate analysis, including NLR, d-NLR, PLR, CRP to lymphocyte ratio, and CRP to albumin ratio [12]. The present study involved a large national multicenter cohort of 10,575 hospitalized COVID-19 patients, aiming to validate the potential association between DLR and short-term mortality. In this national cohort, 10.5% of patients were admitted to the ICU, while in other series, this may reach up to 5–17%. In addition, in this study, ICU mortality was 24.7% compared to up to 40–50% previously published in the literature. Thus, new biomarkers such as DLR might be helpful for a better prognosis prediction [2,3,4,9]. Among all laboratory biomarkers evaluated in this retrospective national cohort that involved 10,575 COVID-19 patients, we found that DLR showed an independent association for in-hospital mortality, proving its potential usefulness as a biomarker of poor prognosis with an optimal cut-off above 1 (adjusted OR 2.12, 95% CI 1.119–4.08) regarding age, ARDS, lymphopenia < 800 µL^−1^, D-dimer > 1000 ng/mL, among others (Table 2), with the worst survival rate for DLR > 1 (HR 2.24; 95% CI 2.03–2.47). In this large multicenter cohort, no other laboratory indices showed an independent association in multivariate analysis.

Hence, in this large national cohort study, we successfully achieved the outcome and validated the usefulness of the novel laboratory index known as DLR for predicting mortality. DLR appears to be a useful prognostic biomarker in the assessment of mortality in hospitalized COVID-19 patients; further, we hypothesize that DLR might be a useful biomarker for other infectious or inflammatory diseases. However, additional prospective studies will be necessary to incorporate it into daily clinical practice.

Our study has some limitations. Firstly, it is a retrospective observational study based on a national dataset. Secondly, all laboratory biomarkers were measured only once at admission; the dynamic changes in the biomarkers were not assessed, and virus load or virus variant was not assessed. Thirdly, our study included patients from March 2020 to March 2022, without differentiation between COVID-19 pandemic waves. Fourthly, patients with pre-existing hematological disorders were excluded, so the results might not apply to this population. Despite these limitations, our study has several strengths: It was a large multicenter study that included all consecutive patients, minimizing selection biases. The role of ratios has been analyzed in the context of real clinical practice, and efforts have been made to minimize information biases when establishing the definitions of the concepts in the methods.

## 5. Conclusions

In summary, this large national multicenter study validates the potential usefulness of the novel laboratory index called the D-dimer to lymphocyte ratio as a prognostic biomarker for in-hospital mortality, measured at admission. Although DLR would be an easy-to-use, accessible, and cost-effective biomarker for daily clinical practice, additional prospective studies will be necessary to incorporate it into daily clinical practice.

## Figures and Tables

**Table 1:** Baseline characteristics and outcomes in the studied population, comparing survivors and non-survivors.

Variable	Total (*n* = 10,575)	Survivors (*n* = 8921)	Non-Survivors (*n* = 1654)	*p* Value
Age, years (mean, SD)	66.9 (±16)	64.8 (±16)	78.2 (±11)	<0.01
Sex male, *n* (%)	6202 (58.6)	5162 (57.9)	1040 (62.9)	<0.01
BMI, kg/m^2^ (mean, SD)	29 (±5)	29 (±5)	30 (±5)	<0.01
Hypertension, *n* (%)	5474 (51.8)	4297 (48.2)	1177 (71.2)	<0.01
Dyslipidemia, *n* (%)	4259 (40.3)	3389 (38.0)	870 (52.6)	<0.01
Heart failure, *n* (%)	691 (6.5)	440 (4.9)	251 (15.2)	<0.01
COPD, *n* (%)	736 (7.0)	529 (5.9)	207 (12.5)	<0.01
Dementia, *n* (%)	815 (7.7)	514 (5.8)	301 (18.2)	<0.01
Cancer, *n* (%)	557 (5.3)	423 (4.7)	134 (8.1)	<0.01
Pneumonia, *n* (%)	4525 (42.8)	3635 (40.7)	890 (53.8)	<0.01
SBP < 100 mmHg, *n* (%)	776 (7.5)	586 (6.7)	190 (11.6)	<0.01
Heart rate > 100 bpm, *n* (%)	2274 (21.8)	1904 (21.6)	370 (22.8)	0.29
Oxygen saturation < 90%, *n* (%)	1937 (18.8)	1247 (14.3)	690 (42.6)	<0.01
ARDS, *n* (%)	3459 (32.7)	2218 (24.9)	1241 (75.0)	<0.01
Laboratory findings				
Hemoglobin, mg/dL (median, P25–P75)	13.7 (12.4–14.9)	13.8 (12.6–14.9)	13.2 (11.6–14.6)	<0.01
Leukocytes, µL^−1^ (median, P25–P75)	6400 (4800–8560)	6300 (4710–8300)	7150 (5290–10,172)	<0.01
Neutrophils, µL^−1^ (median, P25–P75)	4680 (3270–6700)	4520 (3200–6400)	5655 (3800–8455)	<0.01
Neutrophils, >8000/µL, *n* (%)	1660 (15.7)	1195 (13.4)	465 (28.1)	<0.01
Lymphocytes, µL^−1^ (median, P25–P75)	930 (680–1290)	980 (700–1300)	760 (500–1100)	<0.01
Lymphocytes < 800/µL, *n* (%)	4137 (39.1)	3217 (36.1)	920 (55.6)	<0.01
Platelets, ×1000∙µL^−1^ (median, P25–P75)	193 (149–251)	195 (150–253)	183 (139–235)	<0.01
D-dimer, ng/mL (median, P25-P75)	613 (345–1140)	575 (324–1015)	981 (517–1923)	<0.01
D-dimer > 1000 ng/mL, *n* (%)	3074 (29.1)	2263 (25.4)	843 (51.0)	<0.01
CRP, mg/L (median, P25–P75)	64.6 (22.9–130.8)	58.1 (20.6–119.5)	107.5 (49.6–182.9)	<0.01
CRP > 100 mg/L, *n* (%)	3672 (34.7)	2789 (31.3)	883 (53.4)	<0.01
Procalcitonin, µg/L (median, P25–P75)	0.11 (0.06–0.22)	0.10 (0.06–0.18)	0.22 (0.11–0.60)	<0.01
Albumin, g/dL (median, P25–P75)	3.7 (3.3–4.0)	3.8 (3.4–4.1)	3.4 (3.1–3.7)	<0.01
NLR, (median, P25–P75)	4.9 (3.1–8.3)	4.6 (2.9–7.5)	7.3 (4.4–13.0)	<0.01
d-NLR, (median, P25–P75)	3.1 (2.1–4.9)	2.9 (1.9–4.5)	4.4 (2.8–7.0)	<0.01
PLR, (median, P25–P75)	205 (142–303)	200 (140–292)	239 (159–373)	<0.01
CLR, (median, P25–P75)	0.07 (0.02–0.16)	0.06 (0.02–0.14)	0.14 (0.05–0.28)	<0.01
CAR, (median, P25–P75)	17.7 (6.1–37.2)	15.6 (5.4–33.5)	32.4 (14.4–55.7)	<0.01
DLR, (median, P25–P75)	0.67 (0.34–1.45)	0.60 (0.32–1.24)	1.37 (0.63–3.13)	<0.01
ICU admission, *n* (%)	1106 (10.5)	706 (7.9)	400 (24.2)	<0.01
Orotracheal intubation, *n* (%)	789 (7.5)	435 (4.9)	354 (21.5)	<0.01
NIMV, *n* (%)	923 (8.8)	585 (6.6)	338 (20.6)	<0.01
Outcomes				
Mortality, *n* (%)	1654 (15.6)	-	-	-

ARDS: acute respiratory distress syndrome; BMI: body mass index; COPD: chronic obstructive pulmonary disease; CAR: CRP to serum albumin ratio; CLR: CRP to lymphocyte ratio; DLR: D-dimer to lymphocyte ratio; d-NLR: derivate neutrophil to white blood cells ratio; ICU: intensive care unit; NIMV: non-invasive mechanical ventilation; NLR: neutrophil to lymphocyte ratio; PLR: platelet to lymphocyte ratio; SBP: systolic blood pressure.

**Table 2:** Univariate and multivariate * logistic regression analysis for in-hospital mortality.

Variables	OR ^†^	95% CI		*p* Value	OR ^‡^	95% CI		*p* Value
	Univariate				Multivariate			
Age > 70 years	3.17	2.10	4.87	<0.01	2.63	1.59	4.41	<0.01
Sex (female)	0.67	0.44	1.01	0.06	0.66	0.40	1.06	0.09
Dementia	1.70	0.76	3.43	0.17	-	-	-	-
Hypertension	2.51	1.63	3.96	<0.01	1.43	0.85	2.44	0.18
Dyslipidemia	1.35	0.91	2.00	0.13	-	-	-	-
Heart failure	3.99	2.25	6.87	<0.01	1.92	0.96	3.75	0.06
COPD	2.88	1.65	4.86	<0.01	1.14	0.58	2.20	0.70
Cancer	2.02	0.96	3.89	0.05	2.12	0.89	4.75	0.08
ARDS	14.6	7.9	30.2	<0.01	10.9	5.7	23.5	<0.01
SBP < 100 mmHg	1.85	0.93	3.45	0.06	1.98	0.84	4.39	0.10
Oxygen saturation < 90%	2.78	1.82	4.35	<0.01	1.38	0.81	2.31	0.23
Neutrophils > 8000/µL	1.71	1.01	2.79	0.04	1.02	0.50	2.00	0.97
Lymphocyte < 800/µL	2.78	1.85	4.17	<0.01	1.20	0.65	2.24	0.56
D-dimer > 1000 ng/mL	2.13	1.43	3.17	<0.01	1.03	0.56	1.91	0.92
CRP > 100 mg/L	2.87	1.93	4.30	<0.01	1.48	0.58	4.14	0.44
Albumin < 3.5 g/dL	2.38	1.59	3.57	<0.01	1.39	0.83	2.31	0.21
NLR > 6.5 (µL^−1^/µL^−1^)	3.58	2.39	5.44	<0.01	1.10	0.51	2.38	0.81
d-NLR > 4 (µL^−1^/µL^−1^)	3.58	2.40	5.39	<0.01	1.90	0.96	3.82	0.07
PLR > 290 (µL^−1^/µL^−1^)	1.83	1.23	2.72	<0.01	0.62	0.36	1.08	0.09
CLR > 0.1 (mg/L/µL^−1^)	3.41	2.26	5.25	<0.01	1.45	0.64	3.26	0.37
CAR > 25 (mg/L)/(g/dL)	2.93	1.96	4.43	<0.01	0.82	0.28	2.26	0.71
DLR > 1 (ng/mL/µL^−1^)	3.54	2.34	5.47	<0.01	2.12	1.11	4.08	0.02

ARDS: acute respiratory distress syndrome; COPD: chronic obstructive pulmonary disease; CRP: C-reactive protein; CAR: CRP to serum albumin ratio; CLR: CRP to lymphocyte ratio; DLR: D-dimer to lymphocyte ratio; d-NLR: derivate neutrophil to white blood cells ratio; NLR: neutrophil to lymphocyte ratio; PLR: platelet to lymphocyte ratio; SBP: systolic blood pressure. * The multivariate analysis was performed for variables with a *p*-value < 0.1 in the univariate analysis. ^†^ Crude odds ratio; ^‡^ adjusted odds ratio.

## Data Availability

The data that support the findings of this study are available from the corresponding author upon reasonable request.

## Supplementary Materials

The following supporting information can be downloaded at: https://www.mdpi.com/article/10.3390/v16030335/s1.

## References

[1] Hu B., Guo H., Zhou P., Shi Z.L. (2021). Characteristics of SARS-CoV-2 and COVID-19. Nat. Rev. Microbiol..

[2] Zhu N., Zhang D., Wang W., Li X., Yang B., Song J., Zhao X., Huang B., Shi W., Lu R. (2020). China Novel Coronavirus Investigating and Research Team. A novel coronavirus from patients with pneumonia in China, 2019. N. Engl. J. Med..

[3] Coronavirus COVID-19 Global Cases by the Center for Systems Science and Engineering (CSSE) at Johns Hopkins University. https://coronavirus.jhu.edu.

[4] Wiersinga W.J., Rhodes A., Cheng A.C., Peacock S.J., Prescott H.C. (2020). Pathophysiology, transmission, diagnosis, and treatment of Coronavirus disease 2019 (COVID-19): A review. JAMA.

[5] Chams N., Chams S., Badran R., Shams A., Araji A., Raad M., Mukhopadhyay S., Stroberg E., Duval E.J., Barton L.M. (2020). COVID-19: A Multidisciplinary Review. Front. Public. Health.

[6] Gupta A., Madhavan M.V., Sehgal K., Nair N., Mahajan S., Sehrawat T.S., Bikdeli B., Ahluwalia N., Ausiello J.C., Wan E.Y. (2020). Extrapulmonary manifestations of COVID-19. Nat. Med..

[7] Kox M., Waalders N.J.B., Kooistra E.J., Gerretsen J., Pickkers P. (2020). Cytokine Levels in Critically Ill Patients with COVID-19 and Other Conditions. JAMA.

[8] Alonso-Beato R., Lago-Rodríguez M.-O., López-Rubio M., Gómez-Tórtola A., García-Fernández-Bravo I., Oblitas C.-M., Galeano-Valle F., Demelo-Rodríguez P. (2023). Risk of thrombosis recurrence among patients with COVID-19 and surgery-associated venous thromboembolism. Rev. Clin. Esp..

[9] Wu Z., McGoogan J.M. (2020). Characteristics of and Important Lessons from the Coronavirus Disease 2019 (COVID-19) Outbreak in China: Summary of a Report of 72,314 Cases from the Chinese Center for Disease Control and Prevention. JAMA.

[10] Oblitas C.M., Torres-Do-Rego A., García A.G., Mato-Jimeno V., Alonso Gonzalo L., Luis-García S., Enríquez-Gómez A., Baltasar-López M., Bello-Martínez E. (2022). A retrospective analysis of incidence and severity of COVID-19 among hypertensive patients: The other side. Clin. Exp. Hypertens..

[11] Vardavas C.I., Mathioudakis A.G., Nikitara K., Stamatelopoulos K., Georgiopoulos G., Phalkey R., Leonardi-Bee J., Fernandez E., Carnicer-Pont D., Vestbo J. (2022). Prognostic factors for mortality, intensive care unit and hospital admission due to SARS-CoV-2: A systematic review and meta-analysis of cohort studies in Europe. Eur. Respir. Rev..

[12] Oblitas C.M., Galeano-Valle F., Cuenca-Carvajal C., Piqueras-Ruiz S., Alonso-Beato R., Alejandre-de-Oña Á., Carrascosa-Fernández P., Chacón Moreno A.D., Parra-Virto A., Pérez Sanz M.T. (2022). Evaluation of simple laboratory parameters in SARS-CoV-2 infection: The role of ratios. Infect. Dis..

[13] Pastori D., Cormaci V.M., Marucci S., Franchino G., Del Sole F., Capozza A., Fallarino A., Corso C., Valeriani E., Menichelli D. (2023). A Comprehensive Review of Risk Factors for Venous Thromboembolism: From Epidemiology to Pathophysiology. Int. J. Mol. Sci..

[14] Casas-Rojo J., Antón-Santos J., Millán-Núñez-Cortés J., Lumbreras-Bermejo C., Ramos-Rincón J., Roy-Vallejo E., Artero-Mora A., Arnalich-Fernández F., García-Bruñén J., Vargas-Núñez J. (2020). Clinical characteristics of patients hospitalized with COVID-19 in Spain: Results from the SEMI-COVID-19 Registry. Rev. Clin. Esp..

[15] Berenguer J., Ryan P., Rodríguez-Baño J., Jarrín I., Carratalà J., Pachón J., Yllescas M., Arriba J.R., Muñoz E.A., Gil Divasson P. (2020). COVID-19@Spain Study Group. Characteristics and predictors of death among 4035 consecutively hospitalized patients with COVID-19 in Spain. Clin. Microbiol. Infect.

[16] Elshazli R.M., Toraih E.A., Elgaml A., El-Mowafy M., El-Mesery M., Amin M.N., Hussein M.H., Killackey M.T., Fawzy M.S., Kandil E. (2020). Diagnostic and prognostic value of hematological and immunological markers in COVID-19 infection: A meta-analysis of 6320 patients. PLoS ONE.

[17] Oblitas C.M., Galeano-Valle F., Ramírez-Navarro J., López-Cano J., Monterrubio-Manrique Á., García-Gámiz M., Sancho-González M., Arenal-López S., Álvarez-Sala Walther L.A., Demelo-Rodríguez P. (2021). Mid-Regional Pro-Adrenomedullin, Methemoglobin and Carboxyhemoglobin as Prognosis Biomarkers in Critically Ill Patients with COVID-19: An Observational Prospective Study. Viruses.

[18] Zhang H., Wu H., Pan D., Shen W. (2022). D-dimer levels and characteristics of lymphocyte subsets, cytokine profiles in peripheral blood of patients with severe COVID-19: A systematic review and meta-analysis. Front. Med..

[19] Ulloque-Badaracco J.R., Ivan Salas-Tello W., Al-kassab-Córdova A., Alarcón-Braga E.A., Benites-Zapata V.A., Maguiña J.L., Hernandez A.V. (2021). Prognostic value of the neutrophil-to-lymphocyte ratio in COVID-19 patients: A systematic review and meta-analysis. Int. J. Clin. Pract..

[20] Sarkar S., Kannan S., Khanna P., Singh A.K. (2022). Role of platelet-to-lymphocyte count ratio (PLR), as a prognostic indicator in COVID-19: A systematic review and meta-analysis. J. Med. Virol..

[21] Zinellu A., Mangoni A.A. (2022). A systematic review and meta-analysis of the association between the neutrophil, lymphocyte, and platelet count, neutrophil-to-lymphocyte ratio, and platelet-to-lymphocyte ratio and COVID-19 progression and mortality. Expert. Rev. Clin. Immunol..

[22] Uzum Y., Turkkan E. (2023). Predictivity of CRP, Albumin, and CRP to Albumin Ratio on the Development of Intensive Care Requirement, Mortality, and Disease Severity in COVID-19. Cureus.

[23] Giner-Galvañ V., Pomares-Gómez F.J., Quesada J.A., Rubio-Rivas M., Tejada-Montes J., Baltasar-Corral J., Taboada-Martínez M.L., Sánchez-Mesa B., Arnalich-Fernández F., Del Corral-Beamonte E. (2022). On Behalf of The Semi-Covid-Network. C-Reactive Protein and Serum Albumin Ratio: A Feasible Prognostic Marker in Hospitalized Patients with COVID-19. Biomedicines.

[24] Zavalaga-Zegarra H.J., Palomino-Gutierrez J.J., Ulloque-Badaracco J.R., Mosquera-Rojas M.D., Hernandez-Bustamante E.A., Alarcon-Braga E.A., Benites-Zapata V.A., Herrera-Añazco P., Hernandez A.V. (2022). C-Reactive Protein-to-Albumin Ratio and Clinical Outcomes in COVID-19 Patients: A Systematic Review and Meta-Analysis. Trop. Med. Infect. Dis..

