# Supplementary material for: Prognostic Value of D-dimer to Lymphocyte Ratio (DLR) in Hospitalized Coronavirus Disease 2019 (COVID-19) Patients: A Validation Study in a National Cohort
Source: Viruses. 2024 Feb 22;16(3):335. doi: 10.3390/v16030335 (PMC10976262; doi:10.3390/v16030335)
Supplement: Supplementary file 1 [file viruses-16-00335-s001.zip › viruses-2836307-supplementary.pdf]

Supplementary Files

List of the SEMI-COVID-19 Network members

Coordinator of the SEMI-COVID-19 Registry: José Manuel Casas Rojo.

SEMI-COVID-19 Scientific Committee Members: José Manuel Casas Rojo, José Manuel Ramos Rincón, Carlos Lumbreras Bermejo, Jesús Millán Núñez-Cortés, Juan Miguel Antón Santos, Ricardo Gómez Huelgas.

Members of the SEMI-COVID-19 Group

H. Univ. de Bellvitge. L'Hospitalet de Llobregat (Barcelona)

Xavier Corbella, Francesc Formiga Pérez, Narcís Homs, Abelardo Montero, Jose María Mora-Luján, Manuel Rubio-Rivas

H. Costa del Sol. Marbella (Málaga)

Victoria Augustín Bandera, Javier García Alegría, Nicolás Jiménez-García, Jairo Luque del Pino, María Dolores Martín Escalante, Francisco Navarro Romero, Victoria Nuñez Rodriguez, Julián Olalla Sierra

H. U. S. Juan de Alicante (Alicante)

Marisa Asensio Tomás, David Balaz, David Bonet Tur, Ruth Cañizares Navarro, Paloma Chazarra Pérez, Jesús Corbacho Redondo, Eliana Damonte White, María Escamilla Espínola, Leticia Espinosa Del Barrio, Pedro Jesús Esteve Atiénzar, Carles García Cervera, David Francisco García Núñez, Francisco Garrido Navarro, Vicente Giner Galvañ, Angie Gómez Uranga, Javier Guzmán Martínez, Isidro Hernández Isasi, Lourdes Lajara Villar, Verónica Martínez Sempere, Juan Manuel Núñez Cruz, Sergio Palacios Fernández, Juan Jorge Peris García, Rafael Piñol Pleguezuelos, Andrea Riaño Pérez, José Miguel Seguí Ripoll, Azucena Sempere Mira, Philip Wikman-Jorgensen

H. U. 12 de Octubre. Madrid

Paloma Agudo de Blas, Coral Arévalo Cañas, Blanca Ayuso, José Bascuñana Morejón, Samara Campos Escudero, María Carnevali Frías, Santiago Cossio Tejido, Borja de Miguel Campo, Carmen Díaz Pedroche, Raquel Diaz Simon, Ana García Reyne, Laura Ibarra Veganzones, Lucia Jorge Huerta, Antonio Lalueza Blanco, Jaime Laureiro Gonzalo, Jaime Lora-Tamayo, Carlos Lumbreras Bermejo, Guillermo Maestro de la Calle, Rodrigo Miranda Godoy, Barbara Otero Perpiña, Diana Paredes Ruiz, Marcos Sánchez Fernández, Javier Tejada Montes

H. de Cabueñes. Gijón (Asturias)

Ana María Álvarez Suárez, Carlos Delgado Vergés, Rosa Fernandez-Madera Martínez, Eva Mª Fonseca Aizpuru, Alejandro Gómez Carrasco, Cristina Helguera Amezua, Juan Francisco López Caleya, Diego López Martínez, María del Mar Martínez López, Aleida Martínez Zapico, Carmen Olabuenaga Iscar, Lucía Pérez Casado, María Luisa Taboada Martínez, Lara María Tamargo Chamorro

H. U. Gregorio Marañon. Madrid

Laura Abarca Casas, Álvaro Alejandre de Oña, Rubén Alonso Beato, Leyre Alonso Gonzalo, Jaime Alonso Muñoz, Crhistian Mario Amodeo Oblitas, Cristina Ausín García, Marta Bacete Cebrián, Jesús Baltasar Corral, Maria Barrientos Guerrero, Alejandro D. Bendala Estrada, María Calderón Moreno, Paula Carrascosa Fernández, Raquel Carrillo, Sabela Castañeda Pérez, Eva Cervilla Muñoz, Agustín Diego Chacón Moreno, Maria Carmen Cuenca Carvajal, Sergio de Santos, Andrés Enríquez Gómez, Eduardo Fernández Carracedo, María Mercedes Ferreiro-Mazón Jenaro, Francisco Galeano Valle, Alejandra Garcia, Irene Garcia Fernandez-Bravo, María Eugenia García Leoni, María Gómez Antúnez, Candela González San Narciso, Anthony Alexander Gurjian, Lorena Jiménez Ibáñez, Cristina Lavilla Olleros, Cristina Llamazares Mendo, Sara Luis García, Víctor Mato Jimeno, Clara Millán Nohales, Jesús Millán Núñez-Cortés, Sergio Moragón Ledesma, Antonio Muiño Míguez, Cecilia Muñoz Delgado, Lucía Ordieres Ortega, Susana Pardo Sánchez, Alejandro Parra Virto, María Teresa Pérez Sanz, Blanca Pinilla Llorente, Sandra Piqueras Ruiz, Guillermo Soria Fernández-Llamazares, María Toledano Macías, Neera Toledo Samaniego, Ana Torres do Rego, Maria Victoria Villalba Garcia, Gracia Villarreal, María Zurita Etayo

C. H. U. de Badajoz

Rafael Aragon Lara, Inmaculada Cimadevilla Fernandez, Juan Carlos Cira García, Gema Maria García García, Julia Gonzalez Granados, Beatriz Guerrero Sánchez, Francisco Javier Monreal Periáñez, Maria Josefa Pascual Perez

C. H. U. de Albacete
Jose Luis Beato Pérez, Maria Lourdes Sáez Méndez

H. Royo Villanova. Zaragoza
Nicolás Alcalá Rivera, Anxela Crestelo Vieitez, Esther del Corral Beamonte, Jesús Díez Manglano, Isabel Fiteni Mera, Maria del Mar Garcia Andreu, Martin Gericó Aseguinolaza, Cristina Gallego Lezaun, Claudia Josa Laorden, Raul Martínez Murgui, Marta Teresa Matía Sanz

H. Reg. Univ. de Málaga
Mª Mar Ayala-Gutiérrez, Rosa Bernal López, José Bueno Fonseca, Verónica Andrea Buonaiuto, Luis Francisco Caballero Martínez, Lidia Cobos Palacios, Clara Costo Muriel, Francis de Windt, Ana Teresa Fernandez-Truchaud Christophel, Paula García Ocaña, Ricardo Gómez Huelgas, Javier Gorospe García, José Antonio Hurtado Oliver , Sergio Jansen-Chaparro, Maria Dolores López-Carmona, Pablo López Quirantes, Almudena López Sampalo, Elizabeth Lorenzo-Hernández, Juan José Mancebo Sevilla, Jesica Martín Carmona, Luis Miguel Pérez-Belmonte, Iván Pérez de Pedro, Araceli Pineda-Cantero, Carlos Romero Gómez, Michele Ricci, Jaime Sanz Cánovas

H. U. La Paz. Madrid
Jorge Álvarez Troncoso, Francisco Arnalich Fernández, Francisco Blanco Quintana, Carmen Busca Arenzana, Sergio Carrasco Molina, Aranzazu Castellano Candalija, Germán Daroca Bengoa, Alejandro de Gea Grela, Alicia de Lorenzo Hernández, Alejandro Díez Vidal, Carmen Fernández Capitán, Maria Francisca García Iglesias, Borja González Muñoz, Carmen Rosario Herrero Gil, Juan María Herrero Martínez, Víctor Hontañón, Maria Jesús Jaras Hernández, Carlos Lahoz, Cristina Marcelo Calvo, Juan Carlos Martín Gutiérrez, Monica Martinez Prieto, Elena Martínez Robles, Araceli Menéndez Saldaña, Alberto Moreno Fernández, Jose Maria Mostaza Prieto, Ana Noblejas Mozo, Carlos Manuel Oñoro López, Esmeralda Palmier Peláez, Marina Palomar Pampyn, Maria Angustias Quesada Simón, Juan Carlos Ramos Ramos, Luis Ramos Ruperto, Aquilino Sánchez Purificación, Teresa Sancho Bueso, Raquel Sorriguieta Torre, Clara Itziar Soto Abanedes, Yeray Untoria Tabares, Marta Varas Mayoral, Julia Vásquez Manau

H. Clínico de Santiago de Compostela (A Coruña)
Maria del Carmen Beceiro Abad, Maria Aurora Freire Romero, Sonia Molinos Castro, Emilio Manuel Paez Guillan, María Pazo Nuñez, Paula Maria Pesqueira Fontan

H. U. Reina Sofía. Córdoba
Antonio Pablo Arenas de Larriva, Pilar Calero Espinal, Javier Delgado Lista, Francisco Fuentes-Jiménez, María del Carmen Guerrero Martínez, María Jesús Gómez Vázquez, Jose Jiménez Torres, Laura Limia Pérez, José López-Miranda, Laura Martín Piedra, Marta Millán Orge, Javier Pascual Vinagre, Pablo Pérez-Martinez, María Elena Revelles Vílchez, Angela Rodrigo Martínez, Juan Luis Romero Cabrera, José David Torres-Peña.

C. A. U. de Salamanca
Gloria María Alonso Claudio, Víctor Barreales Rodríguez, Cristina Carbonell Muñoz, Adela Carpio Pérez, María Victoria Coral Orbes, Daniel Encinas Sánchez, Sandra Inés Revuelta, Miguel Marcos Martín, José Ignacio Martín González, José Ángel Martín Oterino, Leticia Moralejo Alonso, Sonia Peña Balbuena, María Luisa Pérez García, Ana Ramon Prados, Beatriz Rodríguez-Alonso, Ángela Romero Alegría, Maria Sanchez Ledesma, Rosa Juana Tejera Pérez

H. Universitario Dr. Peset. Valencia
Juan Alberto Aguilera Ayllón, Arturo Artero, María del Mar Carmona Martín, María José Fabiá Valls, Maria de Mar Fernández Garcés, Ana Belén Gómez Belda, Ian López Cruz, Manuel Madrazo López, Elisabeth Mateo Sanchis, Jaume Micó Gandia, Laura Piles Roger, Adela Maria Pina Belmonte, Alba Viana García

H. U. Puerta de Hierro. Madrid
Ane Andrés Eisenhofer, Ana Arias Milla, Isolina Baños Pérez, Laura Benítez Gutiérrez, Javier Bilbao Garay, Jorge Calderón Parra, Alejandro Callejas Díaz, Erika Camacho Da Silva, M ª Cruz Carreño Hernández, Raquel Castejón Díaz, María Jesús Citores Sánchez, Carmen Cubero Gozalo, Valentín Cuervas-Mons Martínez, Laura Dorado Doblado, Sara de la Fuente Moral, Alberto Díaz de Santiago, Itziar Diego Yagüe, Ignacio Donate Velasco, Ana María Duca, Pedro Durán del Campo, Gabriela Escudero López, Esther Expósito Palomo, Ana Fernández Cruz, Amy Galán Gómez, Sonia García Prieto, Beatriz García Revilla, Miguel Ángel García Viejo, Javier Gómez Irusta, Patricia González Merino, Edith Vanessa Gutiérrez Abreu,

Isabel Gutiérrez Martín, Ángela Gutiérrez Rojas, Andrea Gutiérrez Villanueva, Jesús Herráiz Jiménez, Fátima Ibáñez Estéllez, Pedro Laguna del Estal, Mª Carmen Máinez Sáiz, Carmen de Mendoza Fernández, María Martínez Urbistondo, Fernando Martínez Vera, María Mateos Seirul-lo, Susana Mellor Pita, Patricia A. Mills Sánchez, Esther Montero Hernández, Alberto Mora Vargas, Victor Moreno-Torres Concha, Ignacio Morrás De La Torre, Elena Múñez Rubio, Rosa Muñoz de Benito, Alejandro Muñoz Serrano, Pablo Navarro Palomo, Ilduara Pintos Pascual, Arturo José Ramos Martín-Vegue, Antonio Ramos Martínez, Celia Rodríguez Olleros, Alberto Roldán Montaud, Yolanda Romero Pizarro, Silvia Rosado García, Diana Ruiz de Domingo, David Sánchez Ortiz, Enrique Sánchez Chica, Irene Solano Almena, Elena Suanzes Martin, Yale Tung Chen, Pablo Tutor de Ureta, Ángela Valencia Alijo, Jose Manuel Vázquez Comendador, Juan Antonio Vargas Núñez.

H. Clínico San Carlos. Madrid
Inés Armenteros Yeguas, Javier Azaña Gómez, Julia Barrado Cuchillo, Irene Burruezo López, Noemí Cabello Clotet, Alberto E. Calvo Elías, Elpidio Calvo Manuel, Carmen María Cano de Luque, Cynthia Chocron Benbunan, Laura Dans Vilan, Claudia Dorta Hernández, Ester Emilia Dubon Peralta, Vicente Estrada Pérez, Santiago Fernandez-Castelao, Marcos Oliver Fragiel Saavedra, José Luis García Klepzig, Maria del Rosario Iguarán Bermúdez, Esther Jaén Ferrer, Alejandro Maceín Rodríguez, Alejandro Marcelles de Pedro, Rubén Ángel Martín Sánchez, Manuel Méndez Bailón, Sara Miguel Álvarez, Maria José Nuñez Orantos, Carolina Olmos Mata, Eva Orviz García, David Oteo Mata, Cristina Outon González, Juncal Perez-Somarriba, Pablo Pérez Mateos, Maria Esther Ramos Muñoz, Xabier Rivas Regaira, Laura Mª Rodríguez Gallardo, Iñigo Sagastagoitia Fornie, Alejandro Salinas Botrán, Miguel Suárez Robles, Maddalena Elena Urbano, Andrea María Vellisca González, Miguel Villar Martínez

H. G. U. de Elda (Alicante)
Carmen Cortés Saavedra, Jennifer Fernández Gómez, Borja González López, María Soledad Hernández Garrido, Ana Isabel López Amorós, Santiago López Gil, Maria de los Reyes Pascual Pérez, Nuria Ramírez Perea, Andrea Torregrosa García

Complejo Asistencial de Segovia
Daniel Monge Monge, Eva María Ferreira Pasos, Alba Varela García

H. Miguel Servet. Zaragoza
Luis Sáez Comet, Laura Letona Giménez, Uxua Asín Samper, Gonzalo Acebes Repiso, José Miguel García Bruñén, Mónica Llorente Barrio, María Aranzazu Caudevilla Martínez, Jesús Javier González Igual, Rosa García Fenoll

H. U. La Princesa. Madrid
María Aguilera García, Ester Alonso Monge, Jesús Álvarez Rodríguez, Claudia Alvarez Varela, Miquel Berniz Gòdia, Marta Briega Molina, Marta Bustamante Vega, Jose Curbelo, Alicia de las Heras Moreno, Ignacio Descalzo Godoy, Alexia Constanza Espiño Alvarez, Ignacio Fernández Martín-Caro, Alejandra Franquet López-Mosteiro, Gonzalo Galvez Marquez, María José García Blanco, Yaiza García del Álamo Hernández, Clara García-Rayo Encina, Noemí Gilabert González, Carolina Guillamo Rodríguez, Nicolás Labrador San Martín, Manuel Molina Báez, Carmen Muñoz Delgado, Pedro Parra Caballero, Javier Pérez Serrano, Laura Rabes Rodríguez, Pablo Rodríguez Cortés, Carlos Rodriguez Franco, Emilia Roy-Vallejo, Monica Rueda Vega, Aresio Sancha Lloret, Beatriz Sánchez Moreno, Marta Sanz Alba, Jorge Serrano Ballesteros, Alba Somovilla, Carmen Suarez Fernández, Macarena Vargas Tirado, Almudena Villa Marti

H. Infanta Sofía. SS de los Reyes (Madrid)
José Francisco Pascual Pareja, Isabel Perales Fraile, Arturo Muñoz Blanco, Rafael del Castillo Cantero, José Luis Valle López, Isabel Rábago Lorite, Rebeca Fuerte Martínez, Inés Suárez García, Llanos Soler Rangel

H. U. de A Coruña
Alicia Alonso Álvarez, Olaya Alonso Juarros, Ariadna Arévalo López, Carmen Casariego Castiñeira, Ana Cerezales Calviño, Marta Contreras Sánchez, Ramón Fernández Varela, Santiago J. Freire Castro, Ana Padín Trigo, Rafael Prieto Jarel, Fátima Raad Varea, Ignacio Ramil Freán, Laura Ramos Alonso, Francisco Javier Sanmartín Pensado, David Vieito Porto

H. Moisès Broggi. Sant Joan Despí (Barcelona)
Judit Aranda Lobo, Lucía Feria Casanovas, Jose Loureiro Amigo, Miguel Martín Fernández, Isabel Oriol Bermúdez, Melani Pestaña Fernández, Nicolas Rhyman, Nuria Vázquez Piqueras

H. de Pozoblanco (Córdoba)
José Nicolás Alcalá Pedrajas, Antonia Márquez García, Inés Vargas

H. U. Río Hortega. Valladolid
Irene Arroyo Jiménez, Marina Cazorla González, Marta Cobos-Siles, Luis Corral-Gudino, Pablo Cubero-Morais, María González Fernández, José Pablo Miramontes González, Marina Prieto Dehesa, Pablo Sanz Espinosa

H. Nuestra Señora del Prado. Talavera de la Reina (Toledo)
Sonia Casallo Blanco, Jeffrey Oskar Magallanes Gamboa, Cristina Salazar Mosteiro, Andrea Silva Asiain

H. U. Infanta Cristina. Parla (Madrid)
Juan Miguel Antón Santos, Ana Belén Barbero Barrera, Blanca Beamonte Vela, Coralia Bueno Muiño, Charo Burón Fernández, Ruth Calderón Hernáiz, Irene Casado López, José Manuel Casas Rojo, Andrés Cortés Troncoso, Pilar Cubo Romano, Francesco Deodati, Alejandro Estrada Santiago, Gonzalo García Casasola Sánchez, Elena García Guijarro, Francisco Javier García Sánchez, Pilar García de la Torre, Mayte de Guzmán García-Monge, Davide Luordo, María Mateos González, José A. Melero Bermejo, Cruz Pastor Valverde, José Luis Pérez Quero, Fernando Roque Rojas, Lorea Roteta García, Elena Sierra Gonzalo, Francisco Javier Teigell Muñoz, Juan Vicente de la Sota, Javier Villanueva Martínez

H. de Urduliz Alfredo Espinosa (Vizcaya)
Miriam García Gómez, Pablo Ramírez Sánchez, Gorka Arroita Gonzalez, Alazne Lartategi Iraurgi, Asier Aranguren Arostegui, Paula Arriola Martínez, Isabel María Portales Fernández, Esther Martinez Becerro, Amalur Iza Jiménez, Cristian Vidal Núñez, María Aparicio López, Eduardo García López, Mª Soledad Azcona Losada, Beatriz Ruiz Estévez

H. Virgen de la Salud. Toledo
Ana Maria Alguacil Muñoz, Marta Blanco Fernández, Veronica Cano, Ricardo Crespo Moreno, Fernando Cuadra Garcia-Tenorio, Blanca Díaz-Tendero Nájera, Raquel Estévez González, María Paz García Butenegro, Alberto Gato Díez, Verónica Gómez Caverzaschi, Piedad María Gómez Pedraza, Julio González Moraleja, Raúl Hidalgo Carvajal, Patricia Jiménez Aranda, Raquel Labra González, Áxel Legua Caparachini, Pilar Lopez Castañeyra, Agustín Lozano Ancin, Jose Domingo Martin Garcia, Cristina Morata Romero, María Jesús Moya Saiz, Helena Moza Moríñigo, Gemma Muñiz Nicolás, Enriqueta Muñoz Platon, Filomena Oliveri, Elena Ortiz Ortiz, Raúl Perea Rafael, Pilar Redondo Galán, María Antonia Sepulveda Berrocal, Vicente Serrano Romero de Ávila, Pilar Toledano Sierra, Yamilex Urbano Aranda, Jesús Vázquez Clemente, Carmen Yera Bergua

H. de Sagunto (Valencia)
Enrique Rodilla Sala, Jose María Pascual Izuel, Zineb Karroud Zamrani

H. U. Son Llàtzer. Palma de Mallorca
Andrés de la Peña Fernández, Almudena Hernández Milián

H. Santa Marina. Bilbao
María Areses Manrique, Ainara Coduras Erdozain, Ane Labirua-Iturburu Ruiz

H. Juan Ramón Jiménez. Huelva
Francisco Javier Bejarano Luque, Francisco-Javier Carrasco-Sánchez, Mercedes de-Sousa-Baena, Jaime Díaz Leal, Aurora Espinar Rubio, Maria Franco Huertas, Juan Antonio García Bravo, Andrés Gonzalez Macías, Encarnación Gutiérrez Jiménez, Alicia Hidalgo Jiménez, Constantino Lozano Quintero, Carmen Mancilla Reguera, Francisco Javier Martínez Marcos, Francisco Muñoz Beamud, Maria Pérez-Aguilar, Alícia Pérez Jiménez, Virginia Rodríguez Castaño, Alvaro Sánchez de Alcazar del Río, Leire Toscano Ruiz

H. San Pedro. Logroño (La Rioja)
Diana Alegre González, Irene Ariño Pérez de Zabalza, Sergio Arnedo Hernández, Jorge Collado Sáenz, Beatriz Dendariena, Marta Gómez del Mazo, Iratxe Martínez de Narvajas Urra, Sara Martínez Hernández, Estela Menendez Fernández, Jose Luís Peña Somovilla, Elisa Rabadán Pejenaute

H. del Henares. Coslada (Madrid)

Jesús Ballano Rodríguez-Solís, Luis Cabeza Osorio, María del Pilar Fidalgo Montero, Mª Isabel Fuentes Soriano, Erika Esperanza Lozano Rincón, Ana Martín Hermida, Jesús Martínez Carrilero, José Ángel Pestaña Santiago, Manuel Sánchez Robledo, Patricia Sanz Rojas, Nahum Jacobo Torres Yebes, Vanessa Vento

H. U. Ramón y Cajal. Madrid
Luis Fernando Abrego Vaca, Ana Andréu Arnanz, Octavio Arce García, Marta Bajo González, Pablo Borque Sanz, Alberto Cozar Llisto, Sonia de Pedro Baena, Beatriz Del Hoyo Cuenda, Martin Fabregate-Fuente, María Alejandra Gamboa Osorio, Isabel García Sánchez, Andrés González García, Oscar Alberto López Cisneros, Luis Manzano, Miguel Martínez-Lacalzada, Borja Merino Ortiz, Jimena Rey-García, Elisa Riera González, Cristina Sánchez Díaz, Grisell Starita Fajardo, Cecilia Suárez Carantoña, Adrian Viteri-Noël, Svetlana Zhilina Zhilina

H. U. Torrevieja (Alicante)
Julio César Blázquez Encinar

H. HLA Moncloa. Madrid
Carmen Martínez Cilleros, Isabel Jiménez Martínez, Teresa García Delange

C. H. U. Ourense
Raquel Fernández González, Amara Gonzalez Noya, Carlos Hernández Ceron, Isabel Izuzquiza Avanzini, Ana Latorre Diez, Pablo López Mato, Ana María Lorenzo Vizcaya, Daniel Peña Benítez, Milagros María Peña Zemsch, Lucía Pérez Expósito, Marta Pose Bar, Lara Rey González, Laura Rodrigo Lara

H. U. La Fe. Valencia
Dafne Cabañero, María Calabuig Ballester, Pascual Císcar Fernández, Ricardo Gil Sánchez, Marta Jiménez Escrig, Cristina Marín Amela, Laura Parra Gómez, Carlos Puig Navarro, José Antonio Todolí Parra

C. Asistencial de Zamora
Carlota Tuñón de Almeida, María Esther Fraile Villarejo, Victoria Palomar Calvo, Sara Pintos Otero, Beatriz García López, Carlos Aldasoro Frías, Víctor Madrid Romero, Luis Arribas Pérez, Emilia Martínez Velado

H. de Mataró. Barcelona
Raquel Aranega González, Ramon Boixeda, Javier Fernández Fernández, Carlos Lopera Mármol, Marta Parra Navarro, Ainhoa Rex Guzmán, Aleix Serrallonga Fustier

H. Público de Monforte de Lemos (Lugo)
José López Castro, Manuel Lorenzo López Reboiro, Cristina Sardiña González

C. H. U. de Ferrol (A Coruña)
Hortensia Alvarez Diaz, Tamara Dalama Lopez, Estefania Martul Pego, Carmen Mella Pérez, Ana Pazos Ferro, Sabela Sánchez Trigo, Dolores Suarez Sambade, Maria Trigas Ferrin, Maria del Carmen Vázquez Friol, Laura Vilariño Maneiro

H. Alto Guadalquivir. Andújar (Jaén)
Begoña Cortés Rodríguez

H. Infanta Margarita. Cabra (Córdoba)
María Esther Guisado Espartero, Lorena Montero Rivas, Maria de la Sierra Navas Alcántara, Raimundo Tirado-Miranda

H. U. San Agustin. Avilés (Asturias)
Marta Nataya Solís Marquínez, Víctor Arenas García, Demelsa Blanco Suárez, Natalia García Arenas, Paula Martínez García, David Castrodá Copa, Andrea Álvarez García, Jaime Casal Álvarez, María Jose Menéndez Calderón, Raquel García Noriega, María Caño Rubia, Joaquin Llorente García, Luis Trapiella Martínez, José Ferreiro Celeiro, Diego Eduardo Olivo Aguilar, Irene Maderuelo Riesco, Juan Valdés Bécares, Alba Barragán Mateos, Andrés Astur Treceño García

H. Univ. Ntra. Sra. Candelaria. Sta. Cruz de Tenerife
Joaquín Delgado Casamayor, Diego García Silvera, Andrea Afonso Díaz, Carolina Hernández Carballo, Alicia Tejera, María José Monedero Prieto, María Blanca Monereo Muñoz, José Manuel Del Arco Delgado,

Daniel Rodríguez Díaz, Marta Bethencourt Feria, Francisco Javier Herrera Herrera, María de la Luz Padilla Salazar, Rubén Hernández Luis, Eduardo Mauricio Calderón Ledezma, María del Mar López Gámez, Laura Torres Hernández, Sara Castaño Pérez, Selena Gala Aguilera García, Guillermo Castro Gainett, Alba Gómez Hidalgo, Julia Marfil Daza, Marcelino Hayek Peraza

H. U. Virgen del Rocío. Sevilla
Reyes Aparicio Santos, Máximo Bernabeu-Wittel, Santiago Rodríguez Suárez , María Nieto, Luis Giménez Miranda, Rosa María Gámez Mancera, Fátima Espinosa Torre, Carlos Hernandez Quiles, Concepción Conde Guzmán, Juan Delgado de la Cuesta, Jara Eloisa Ternero Vega, María del Carmen López Ríos, Pablo Díaz Jiménez, Bosco Baron Franco, Carlos Jiménez de Juan, Sonia Gutiérrez Rivero, Julia Lanseros Tenllado, Verónica Alfaro Lara, Aurora González Estrada

H. Marina Baixa. Villajoyosa (Alicante)
Javier Ena, José Enrique Gómez Segado

C. Asist. Univ. de León
Ángel Luis Martínez González, Beatriz Vicente Montes, Rosario María García Die, Alberto Muela Molinero, Manuel Martín Regidor, Raquel Rodríguez Díez

H. del Tajo. Aranjuez (Madrid)
Ruth Gonzalez Ferrer, Virginia Gracia Lorenzo, Raquel Monsalvo Arroyo

H. San Juan de la Cruz. Úbeda (Jaén)
Marcos Guzmán García, Francisco Javier Vicente Hernández

H. Torrecárdenas. Almería
Bárbara Hernández Sierra, Luis Felipe Díez García, Iris El Attar Acedo, Carmen Mar Sánchez Cano

H. Dr. José Molina Orosa. Arrecife (Lanzarote)
Virginia Herrero García, Berta Román Bernal

H. Clinic Barcelona
Júlia Calvo Jiménez, Emmanuel Coloma Bazán, Aina Capdevila Reniu, Joan Ribot Grabalosa, Joaquim Fernández Solà, Irene Carbonell De Boulle, Cristina Gabara Xancó, Olga Rodríguez Núñez

H. Insular de Gran Canaria. Las Palmas G. C.
Carlos Jorge Ripper

H. U. Marqués de Valdecilla. Santander
Marta Fernández-Ayala Novo, José Javier Napal Lecumberri, Nuria Puente Ruiz, Jose Riancho, Isabel Sampedro García

H. General Defensa. Zaragoza
Anyuli Gracia Gutiérrez, Leticia Esther Royo Trallero

H. U. Virgen de las Nieves. Granada
Pablo Conde Baena, Joaquín Escobar Sevilla, Laura Gallo Padilla, Patricia Gómez Ronquillo, Pablo González Bustos, María Navío Botías, Jessica Ramírez Taboada, Mar Rivero Rodríguez

H. U. C. de Asturias. Oviedo
Víctor Asensi Alvarez, Noelia Morán Suárez, Sara Rodríguez Suárez, Silvia Suárez Díaz, Lucia Suárez Pérez, Maria Folgueras Gómez, Claudia Moran Castaño, Lucía Meijide Rodríguez, Carlos Vázquez, Itxasne Cabezón Estévanez, Carmen Yllera Gutiérrez, Maria Martinez Sela

H. Valle del Nalón. Riaño-Langreo (Asturias)
Sara Fuente Cosío, César Manuel Gallo Álvaro, Julia Lobo García, Antía Pérez Piñeiro

H. U. Severo Ochoa. Leganés (Madrid)

Yolanda Casillas Viera, Lucía Cayuela Rodríguez, Carmen de Juan Alvarez, Gema Flox Benitez, Laura García Escudero, Juan Martin Torres, Patricia Moreira Escriche, Susana Plaza Canteli, M Carmen Romero Pérez

H. G. U. de Castellón
Jorge Andrés Soler, Marián Bennasar Remolar, Alejandro Cardenal Álvarez, Daniela Díaz Carlotti, María José Esteve Gimeno, Sergio Fabra Juana, Paula García López, María Teresa Guinot Soler, Daniela Palomo de la Sota, Guillem Pascual Castellanos, Ignacio Pérez Catalán, Celia Roig Martí, Paula Rubert Monzó, Javier Ruiz Padilla, Nuria Tornador Gaya, Jorge Usó Blasco

H. Francesc de Borja. Gandía (Valencia)
M Angeles Martinez Pascual, Leyre Jorquer Vidal

H. de Palamós (Girona)
Ana Alberich Conesa, Mari Cruz Almendros Rivas, Miquel Hortos Alsina, José Marchena Romero, Anabel Martin-Urda Diez-Canseco

H. Platón. Barcelona
Ana Suárez Lombraña

H. U. del Vinalopó. Elche (Alicante)
Francisco Amorós Martínez, Erika Ascuña Vásquez, José Carlos Escribano Stablé, Adriana Hernández Belmonte, Ana Maestre Peiró, Raquel Martínez Goñi, M. Carmen Pacheco Castellanos, Bernardino Soldan Belda, David Vicente Navarro

H. U. del Sureste. Arganda del Rey (Madrid)
Jon Cabrejas Ugartondo, Ana Belén Mancebo Plaza, Arturo Noguerado Asensio, Bethania Pérez Alves, Natalia Vicente López

H. Parc Tauli. Sabadell (Barcelona)
Francisco Epelde, Isabel Torrente

H. U. Quironsalud Madrid
Pablo Guisado Vasco, Ana Roda Santacruz, Ana Valverde Muñoz

H. Santa Bárbara. Soria
Marta León Téllez

H. Virgen de los Lirios. Alcoy (Alicante)
Mª José Esteban Giner

H. San Pedro de Alcántara. Cáceres
Eva García Sardón, Javier Galán González, Luis Gámez Salazar, Angela Agea Garcia, Itziar Montero Días, Alvaro Santaella Gomez, Marta Correa Matos, Selene Núñez Gaspar, Antonio González Nieto

H. Asepeyo Coslada (Madrid)
Alejo Erice Calvo-Sotelo

H. U. Lucus Augusti. Lugo
Raquel Gómez Méndez, Ana Rodríguez Álvarez

H. U. de Canarias. Sta. Cruz de Tenerife
Onán Pérez Hernández, Alina Pérez Ramírez, María Candelaria Martín González, Miguel Nicolas Navarrete Lorite, Lourdes González Navarrete, Julio Cesar Alvisa Negrin, José Fernando Armas González, Iballa Jiménez, Paula Ortega Toledo, Esther Martin Ponce

H. Clínico Universitario de Valladolid
Xjoylin Teresita Egües Torres, Sara Gutiérrez González, Cristina Novoa Fernández, Pablo Tellería Gómez

H. Comarcal de Blanes (Girona)

Oriol Alonso Gisbert, Mercé Blázquez Llistosella, Pere Comas Casanova, Angels Garcia Flores, Anna Garcia Hinojo, Ana Inés Méndez Martínez, Maria del Carmen Nogales Nieves, Agnés Rivera Austrui, Alberto Zamora Cervantes

H. do Salnes. Vilagarcía de Arousa (Pontevedra)
Vanesa Alende Castro, Ana María Baz Lomba, Ruth Brea Aparicio, Marta Fernández Morales, Jesús Manuel Fernández Villar, María Teresa López Monteagudo, Cristina Pérez García, Lorena Rodríguez Ferreira, Diana Sande Llovo, Maria Begoña Valle Feijoo

H. de Poniente. El Ejido (Almería)
Juan Antonio Montes Romero, Jose Luis Serrano Carrillo de Albornoz, Manuel Jesus Soriano Pérez, Encarna Sánchez Martín

H. Virgen del Mar. Madrid
Thamar Capel Astrua, Paola Tatiana Garcia Giraldo, Maria Jesús González Juárez, Victoria Marquez Fernandez, Ada Viviana Romero Echevarry

H. U. HM Montepríncipe
José F. Varona Arche

H. de Montilla (Córdoba)
Adrián Montaño Martínez

H. Infanta Elena. Huelva
María Gloria Rojano Rivero

H. Sierrallana. Torrelavega (Cantabria)
Reina Valle Bernad, Cristina Limia, Cristina Amado Fernández, Andrea Tejero Fernández, Lucia Paz Fajardo, Tomás de Vega Santos

H. de la Axarquía. Vélez-Málaga (Málaga)
Antonio López Ruiz

H. Quironsalud A Coruña
Hector Meijide Míguez
